# Simulated eye height impacts size perception differently depending on real-world posture

**DOI:** 10.1038/s41598-023-47364-6

**Published:** 2023-11-16

**Authors:** Fatemeh Ghasemi, Laurence R. Harris, Björn Jörges

**Affiliations:** Center for Vision Research, York University, 4700 Keele Street, Toronto, ON M3J 1P3 Canada

**Keywords:** Human behaviour, Object vision

## Abstract

Changes in perceived eye height influence visually perceived object size in both the real world and in virtual reality. In virtual reality, conflicts can arise between the eye height in the real world and the eye height simulated in a VR application. We hypothesized that participants would be influenced more by variation in simulated eye height when they had a clear expectation about their eye height in the real world such as when sitting or standing, and less so when they did not have a clear estimate of the distance between their eyes and the real-life ground plane, e.g., when lying supine. Using virtual reality, 40 participants compared the height of a red square simulated at three different distances (6, 12, and 18 m) against the length of a physical stick (38.1 cm) held in their hands. They completed this task in all combinations of four real-life postures (supine, sitting, standing, standing on a table) and three simulated eye heights that corresponded to each participant’s real-world eye height (123cm sitting; 161cm standing; 201cm on table; on average). Confirming previous results, the square’s perceived size varied inversely with simulated eye height. Variations in simulated eye height affected participants’ perception of size significantly more when sitting than in the other postures (supine, standing, standing on a table). This shows that real-life posture can influence the perception of size in VR. However, since simulated eye height did not affect size estimates less in the lying supine than in the standing position, our hypothesis that humans would be more influenced by variations in eye height when they had a reliable estimate of the distance between their eyes and the ground plane in the real world was not fully confirmed.

## Introduction

Estimating the size of objects accurately is important in everyday life, for example, when we are trying to imagine if the couch we are about to buy fits its spot in the living room or when we estimate how to grasp a glass on the other side of the table, and it is strongly linked to the perception of distance^1–4^. Among several visual^5–7^ and non-visual^8, 9^ cues to size and distance, the observer’s eye-height plays a special role in scaling the world relative to the observer’s body^10–15^. Thus, eye height is increasingly important to consider as virtual reality (VR) becomes more widely available: VR allows the free manipulation of eye height from flying a plane to being a mouse, but at the same time a person is aware that they are merely sitting on a chair in their home. Here we investigate potential interactions between the simulated visual eye height in VR and the representation of the observer’s actual eye height in the real world.

In order to understand the influence of eye height on size perception, it is helpful to disentangle the relationship between physical size and size in visual space^16^ in geometrical terms. Object size is constant in physical space, while the object’s retinal size varies depending on the object's distance and the viewer’s eye height. Physical size, retinal size and distance have a relatively simple relationship when the line of sight hits the object orthogonally:

1 $${\text{retinal size}} \left(^\circ \right) = 2*{\text{atan}}\left( {\frac{{0.5*{\text{physical size}}}}{{{\text{distance}}}}} \right)$$

**Figure 1:**
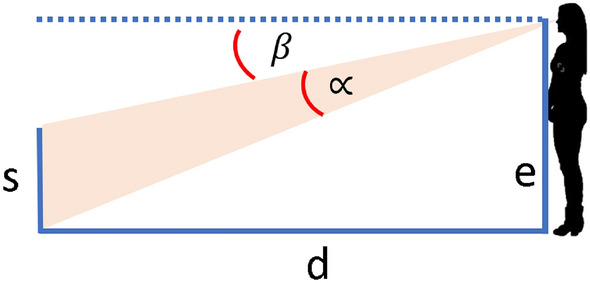
The relationship between eye height (e), distance (d), physical size (s), retinal size ( ∝), and the angle between the horizon and the bottom of an object resting on the floor ($\upbeta$).

The relationship gets more complicated when the object is not located at eye height (see Fig. 1) or is not orthogonally aligned with the line of sight, both in terms of human performance^17^ and the geometry:

2 $${\text{retinal size }}\left(^\circ \right) = 90^\circ - {\text{atan}}\left( {\frac{{{\text{eye height}} - {\text{physical size}}}}{{{\text{distance}}}}} \right) - {\text{atan}}\left( {\frac{{{\text{distance}}}}{{{\text{eyeheight}}}}} \right)$$

Please note that this equation produces very similar results to Eq. (1) when the distance is far bigger than eye height. We can solve this equation for physical size to obtain a potential way to estimate the size of an object given its retinal size, its perceived distance, and the observer’s eye height:

3 $${\text{Physical size }}\left( m \right) = - \tan \left( {90^\circ - {\text{atan}}\left( {\frac{{{\text{distance}}}}{{{\text{eyeheight}}}}} \right) - {\text{retinal size}}} \right)*{\text{distance}} + {\text{eyeheight}}$$

**Figure 2:**
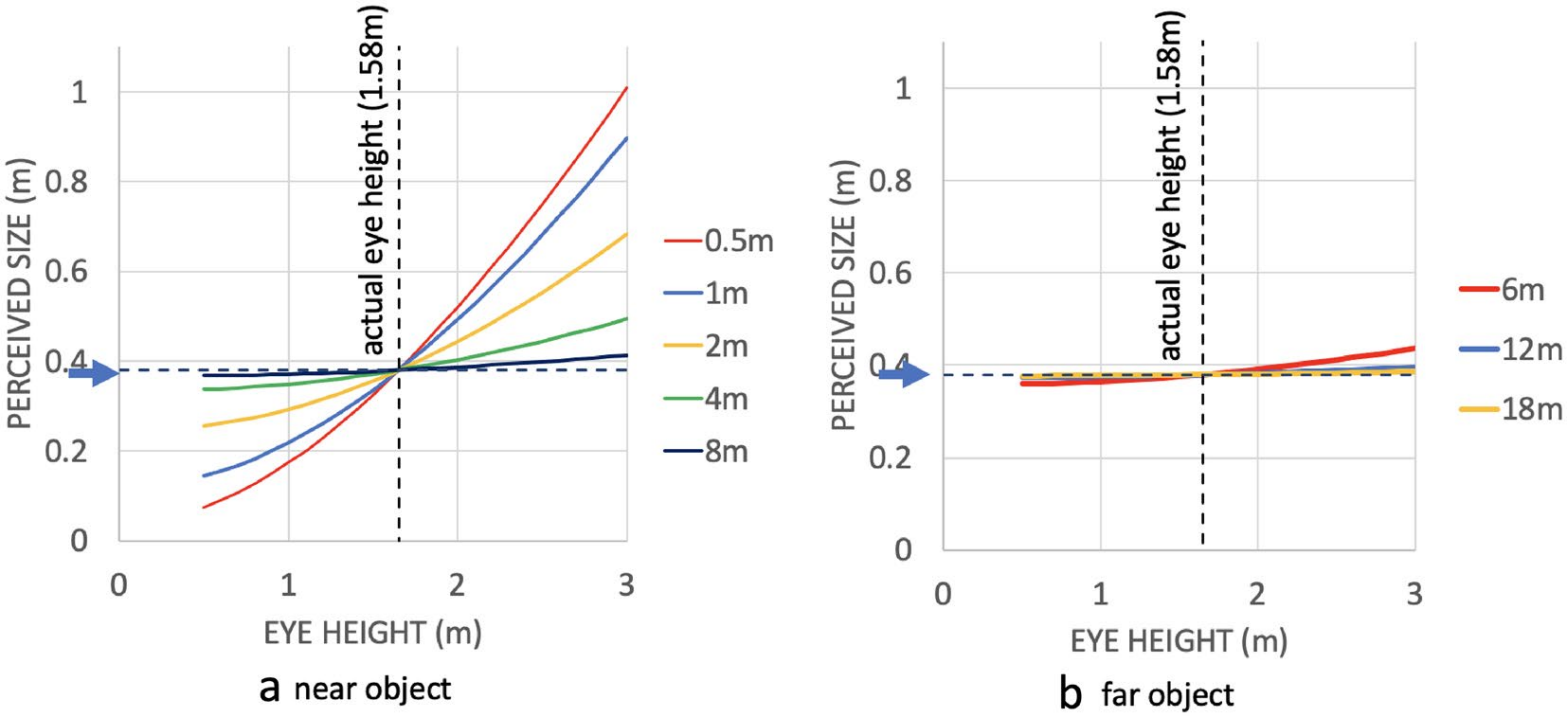
Perceived Size (y axis) as a function of perceived eye height (x axis) for close objects (**a**) and far objects (**b**). We computed the visual angle for each object (as per Eq. 1) and then simulated the perceived size for a range of (mis-)perceived eye-heights (x axis) (as per Eq. 3), while keeping the perceived distance to the target constant at the accurate value. Vertical dashed line indicates true eye height (1.65m), horizontal dashed line and blue arrow indicate actual object size (38.1 m) as used in our experiments. Such a perceived size change would only occur if observers were to base their size judgements exclusively on Eq. (3). You can view the R script used to generate these values on OSF: https://osf.io/mkxad.

Errors in perceiving or representing eye height can thus affect an object’s perceived physical size. Figure 2 shows that for a real eye height of 1.65m misestimations of eye height when viewing objects beyond about 5m have very little effect on perceived size, even when misestimating eye height by a meter. Closer objects, however, could become perceptually grossly distorted if their size was obtained purely using a process described by Eq. (3). It is important to note that these simulations were conducted assuming that distance was perceived accurately. However, when trying to recover distance to an object set on the floor from purely visual information, the potentially least-noisy option is given by multiplying eye-height by the tangent of the elevation angle to where the object touches the ground ($\beta$):

4 $${\text{Distance }}\left( m \right) \, = {\text{ eye}} - {\text{height }}*{\text{ tan }}\left( \beta \right)$$

Since other visual cues to distance like disparity, and physiological cues like accommodation or vergence become extremely noisy for anything but the shortest distances^7, 18^, biases in perceived eye height are therefore likely to lead to corresponding misestimations of distance at such a range. It is therefore, in turn, also unlikely that this is the route by which known biases in perceived size due to changes in eye height are caused.

A more promising visual strategy for determining object size might be what has been called the “horizon ratio”^17,19,20^. For objects resting on the floor, the ratio of the visual angle subtended by the object ($\alpha$) and the angle between the lower edge of the object and the horizon ($\beta )$ is equal to the ratio between the size of the object and the observer’s eye height:

5 $$\frac{\alpha }{\beta } = \frac{{{\text{size}}}}{{\text{eye height}}}$$

This allows judging the size of an object resting on the floor without requiring an estimate of its distance. The ready availability of the cues required would therefore make this the prime strategy, while other cues, such as perceived distance (see Eqs. 3 and 4), may play a role under special circumstances such as when they available to the observer in particularly reliable manner.

**Figure 3:**
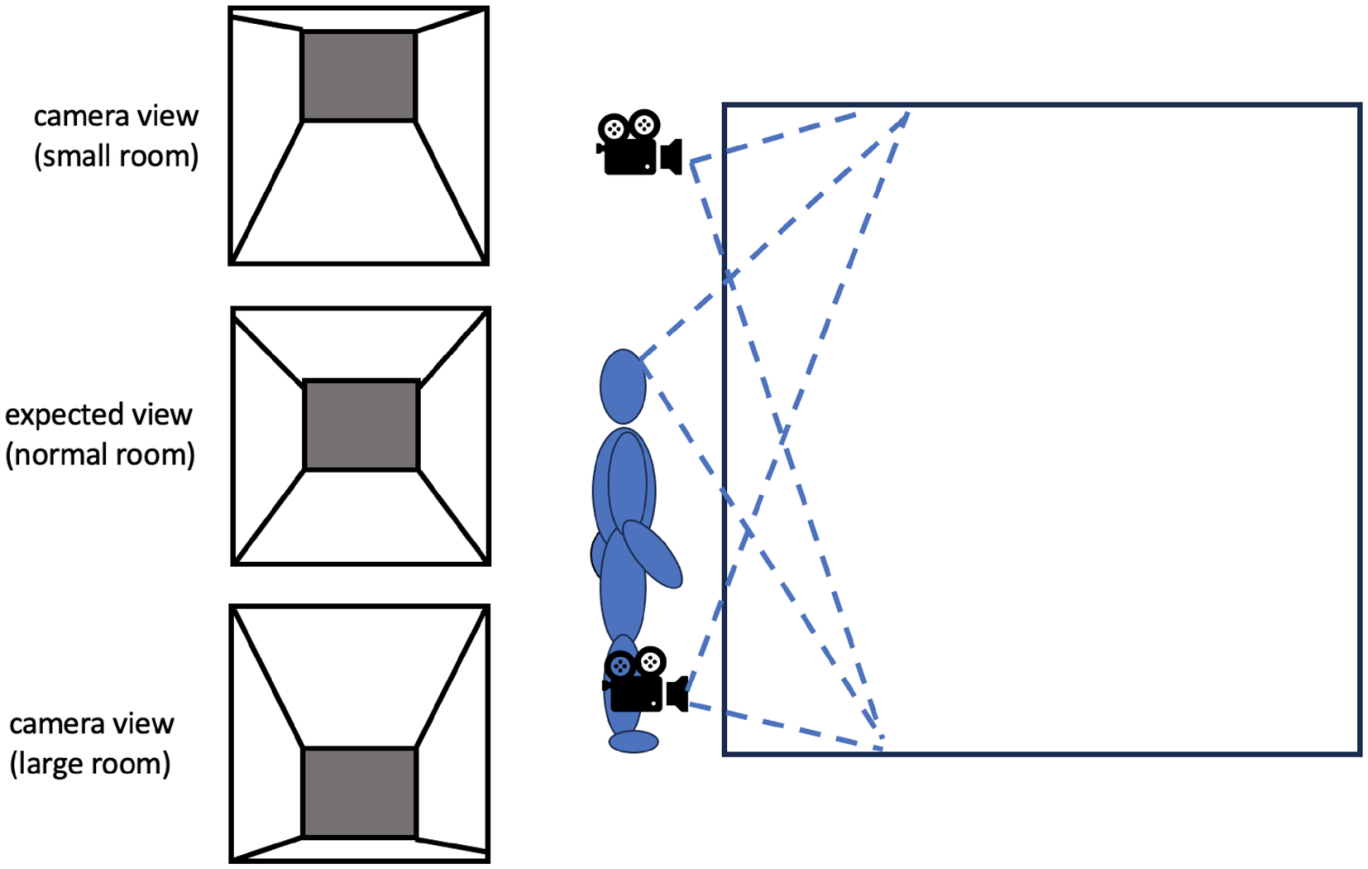
The scaling effect of camera position within a (standard size) room.

Another way that perceived eye height can affect perceived size, especially in virtual environments, is by providing a scaling factor^10,11,15^: Participants may assume that their eye height in the virtual environment is approximately as high above the ground as it would be when adopting their current posture in the real world, and then use this to interpret the scale of their virtual environment: an observer of average height whose viewpoint is simulated as close to the ceiling of a virtual room (Fig. 3) is likely to perceive the ceiling of the room as quite low and the room as fairly small. Objects within it would then also seem smaller. Vice-versa, simulating a low viewpoint would make the room—and with it the objects in it—seem much bigger.

While errors in perceived eye height may occur in real-life situations, they are much more likely to occur in virtual reality, where the many cues that are normally used to estimate eye height (such as accommodation, vergence, or previous experience with the world) are less reliable, ambiguous, or even absent. Our previous experience interacting with the real world with our personal idea of our eye height is thus likely to carry over into VR experiences.

Two studies^12,21^ have investigated the interplay between eye height in the real world and the effect of perceived eye height for the perception of size and/or distance in virtual environments. Leyrer and colleagues^12^ had participants judge the distance to virtual targets using verbal reports of absolute distance. Participants were either sitting, standing upright, or lying prone while facing forwards (i.e., their line of sight was parallel to the ground), and different virtual eye heights were simulated. The observed distance judgements revealed that participants relied more on their real-world eye height in each of these postures than on the simulated visual eye heights. The participants in the von Castell et al. study^21^ judged the size of a virtual room. Similar to Leyrer et al., von Castell and colleagues manipulated the participants’ real-world posture (sitting and standing) and the simulated eye height in VR (corresponding to the participants’ sitting and standing eye height in the real-world but for either corresponding or mismatched postures). Generally, their results were more consistent with participants using visually simulated eye height to estimate the room size rather than their external (actual) eye height. Further, when presented with congruent trials (i.e., visual eye height matched postural eye height) first, participants used both postural and visual eye height. On the flipside, participants relied practically only on their visual eye height when, in their first condition, they were presented with incongruent simulated and real-world eye heights.

Notably, for all the experiments included in these two studies, the participants’ line-of-sight was parallel to the ground plane, that is, they had a clearly defined real-world eye height. Participants would be expected to rely less on simulated eye height in these scenarios than when the relation between line-of-sight and real-world ground plane was less clearly defined, such as lying supine while facing upwards. In such a scenario it would be easier for them to discard their meaningless real-world eye height and rely exclusively on simulated eye height. It is further likely that humans have quite strong (i.e., precise) representations of the eye heights they commonly experience (such as when sitting or standing). Putting participants in an unusual position in the real-world (e.g., by having them stand on a table) might therefore make them discard external eye height information more readily and rely more on the simulated eye height, similar to what we expect for a supine posture.

If participants use their internalized, personal eye height to interpret the size and distance of objects that they see (in real or simulated environments) we hypothesized that varying people’s known eye height above the physical ground before they were immersed in a virtual world may influence how simulated eye height in that environment was interpreted. More specifically, we hypothesized that, in line with previous results^12^, higher simulated eye heights in virtual reality would yield smaller size estimates (corresponding to larger distance estimates in the case of Leyrer and colleagues). We further hypothesized that variation in simulated eye height would affect participants more when they did not have a clear reference for their eye height in the real-world (e.g., when they were lying supine) or when their real-world eye height was not commonly experienced (e.g., when they were standing on a table) than when they did have a clear reference in a common posture, such as when they were sitting upright or standing on the floor.

## Methods

### Participants

46 undergraduate students participated in this study, 6 of whom were excluded due to errors in the data collection or random responses, for a final participant count of 40 (20 men and 20 women; mean age of 21.6 years; SD = 6.2 years). They were recruited from York University’s research pool and received course credit for their participation. All participants gave their written informed consent. This study was approved by the York University ethics committee and conducted in accordance with the Declaration of Helsinki.

### Apparatus

We used a VIVE Pro Eye VR headset (with a visible field of view of 98° vertical and 98° horizontal, a binocular overlap of 90.46° and a resolution of 1440 × 1600 per eye) to present the stimulus, which was programmed in Unity (2021.3.6f1). Participants gave their responses (bigger, smaller) via a finger mouse. We used four postures: sitting on a chair, standing on the floor, standing on a table (height 40 cm) or lying supine on a bed. Participants made their judgements of object size relative to an aluminum stick of 38.1 cm length that they held vertically along their body in their hands.

### Stimuli

**Figure 4:**
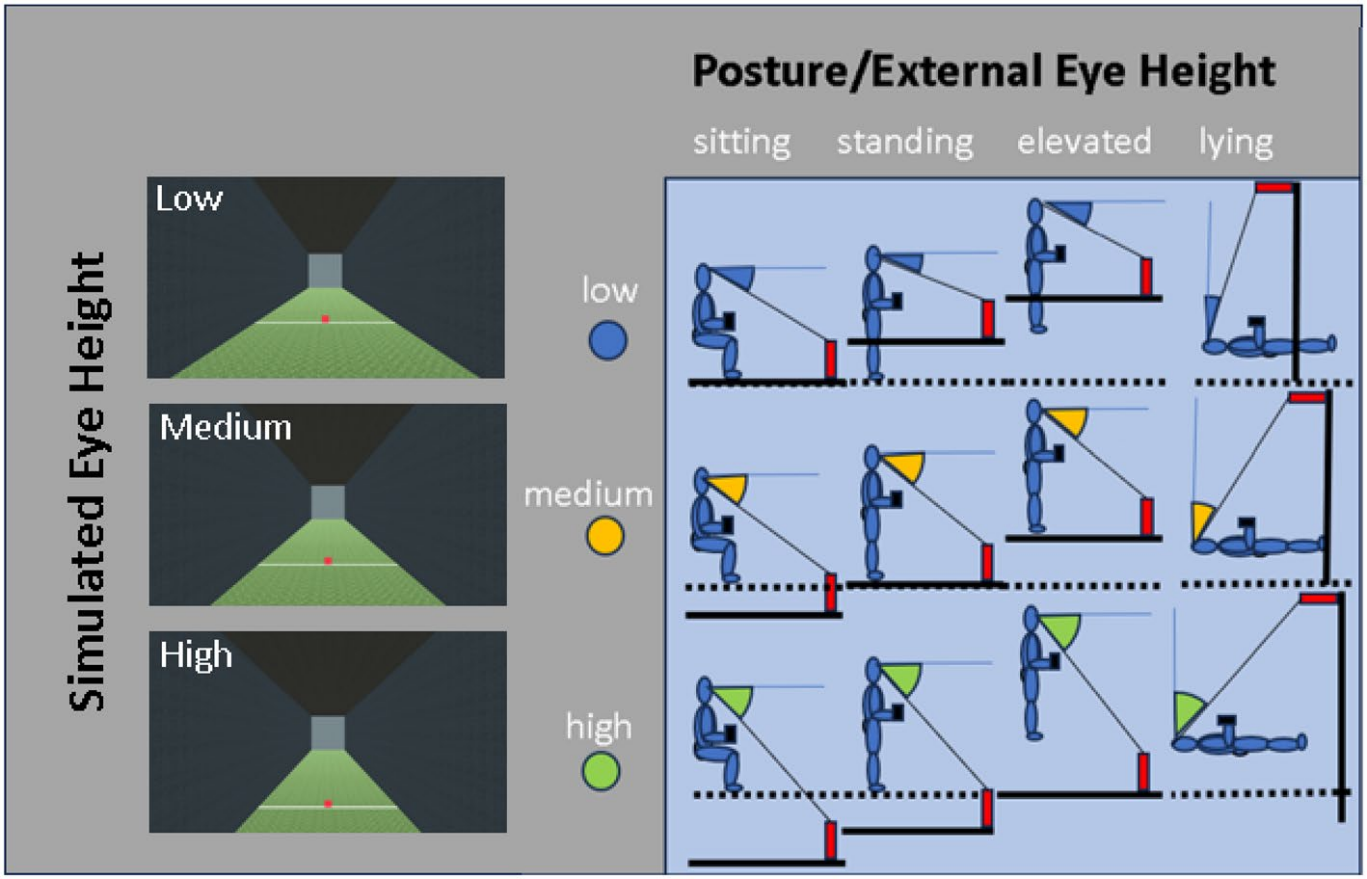
On the right are the combinations of simulated eye height and different postures (12 conditions in total). The solid black line indicates the simulated ground plane, while the dashed line indicates the real-world floor. On the left are screenshots of what participants actually saw for a target at the medium distance of 12m seen from a low eye height (123cm), a medium eye height (161cm) and a high eye height (201cm).

Participants were shown a red square in a VR corridor environment (see Fig. 4), which was simulated at one of three distances (6, 12, and 18 m). Participants were asked to compare the height of the square against the length of a physical aluminum stick of 38.1 cm in their hands. The height of the square was governed by a PEST staircase^22^. We used two staircases for each distance and postural condition, with one staircase starting with the square at a height of 0.2 m and another starting at 2 m. We expected these values to be well below/above the perceived size of the target. The resulting 6 staircases per condition were interleaved. The initial step size was 0.6 m and each individual PEST staircase terminated after 15 trials.

Each participant was tested in four postures (lying supine, sitting upright with their feet on the ground, standing on the ground, standing on a 0.4 m high table) and with three simulated eye heights. The eye heights were determined by each participant’s actual physical eye height as measured by a tape measure before the experiment started when they were sitting (mean 125 ± 6.5 cm), standing on the ground (mean 161 ± 9.5cm) and standing on the table (mean 201 ± 9.5 cm). The visual input was always orthogonal to the long body axis, that is, the simulated ground plane was parallel to the real ground plane in the sitting, standing, and elevated postures, and orthogonal to the real ground plane in the lying supine posture. Participants could look around the environment.

The VR corridor was simulated as having a width of 4 m and a height of 4 m. The participants’ position was centered between the two walls, and their eye height in the environment was adjusted according to their actual eye height and the experimental conditions. There was another wall at the end of the corridor at a simulated distance of 50 m from the observer. The walls and the ceiling had a grey tile texture, and the floor had a grass texture (see Fig. 4).

### Procedure

**Figure 5:**
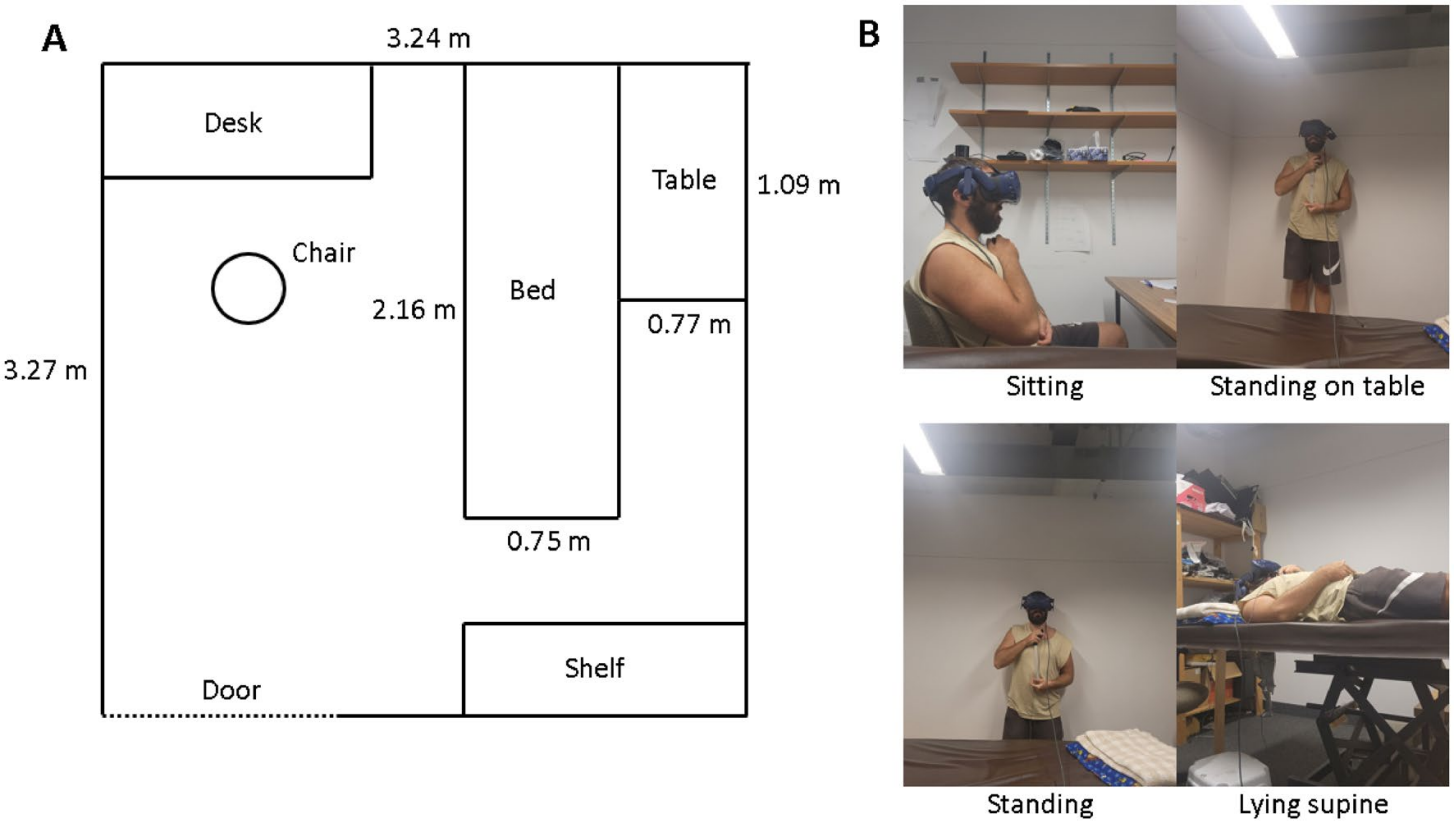
(**A**) Floor plan of the testing room, the overall height being 2.95m. (**B**) Photos of author BJ, who is consenting to publication of these images, in the different postures used in the experiments. Participants held the reference stick in their hands aligned with their long body axis.

After having their eye height measured, participants adopted one of the four postures (see Fig. 5). The sequence of postures was randomized for each participant, and the sequence of simulated eye heights was randomized within each posture. Within each condition, participants were asked to compare the height of a red square (see Fig. 4) to the length of the stick in their hand. They used a finger mouse to report whether they perceived the red square as taller or less tall than the reference stick they held in their hands. Each of the 12 conditions took around 3–5 min (for a total of 35 to 60 min). In the beginning of each block there was some time where the experimenter was setting up the experiment where participants were able to explore the environment with their gaze. However, no such calibration period was formalized as part of the procedure.

### Data analysis

**Figure 6:**
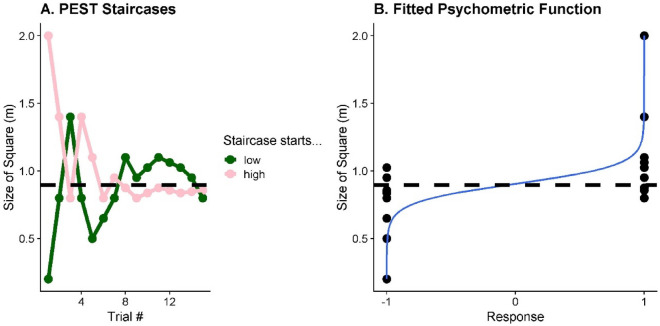
(**A**) Example of two interleaved PEST staircases for one representative participant in one condition. The PEST starting with a large size is plotted in pink and the PEST starting with a small size in green. The dashed black line represents the participant’s PSE as inferred from the logistic function. (**B**) Black circles represent the participant’s response (1 = right for “square taller”, -1 = left for “reference stick longer”) for each presented target size, and the solid blueline is the fitted psychometric function. The dashed black line represents the midpoint of this function, i.e., the participant’s PSE.

We used the data obtained from the staircase procedure to fit psychometric functions. Conventional PEST rules were used to determine the next square’s height to be presented in each staircase^22^ (see Fig. 6A for an example). The initial step size was 0.6m and the initial size was 0.2m for one staircase, and 2m for the other. Each staircase terminated after 15 steps. MATLAB was used to fit the data to a logistic function, where the 50% point represented participants’ perceptual bias—the size at which they were equally likely to choose bigger or smaller (Fig. 6B). The slope of that function (i.e., standard deviation or $\pm$ 34.1% of the bias) was used to represent the perceptual precision (just-noticeable-difference; JND).

We excluded outliers using the mean JNDs (> 0.5) per participant across all conditions as a criterion, with abnormally large JNDs being taken as evidence that the participants either did not understand the task properly, did not pay attention or pressed the buttons randomly. 1 participant was removed using this criterion. We also removed PSEs that were fitted at or extremely close (within 0.05 m) to the boundaries imposed by the fitting algorithm (4 m). This led to exclusion of 14 PSEs from four different participants out of the remaining 1,404 PSEs.

For statistical analysis, we then used Linear Mixed Modelling as implemented in the lme4 package^23^ for R^24^. We used the fitted PSEs as dependent variable, the simulated eye height (as a continuous variable with the values -1 for the lowest eye height, 0 for the medium eye height, and + 1 for the high eye height), the posture (“supine”, “sitting”, “standing”, “elevated”), their interaction, and the simulated target distance (6m, 12m, 18m) as fixed effects, as well as random intercepts and random slopes for the simulated eye height, posture, and distance per participant as random effects:

6 $$\begin{aligned} {\text{PSE }}\sim & {\text{ Simulated Eye Height }}*{\text{ Posture }} + {\text{ Distance }} \\ & + \, \left( {{\text{Simulated Eye height }} + {\text{ Posture }} + {\text{ Distance}}|{\text{ Participant}}} \right) \\ \end{aligned}$$

We used the confint() function from base R to compute bootstrapped 95% confidence intervals to assess statistical significance.

## Results

The Linear Mixed Model we fitted had an intercept of 1.17 m (95% CI = [0.99 m; 1.34 m]). None of comparisons between the postures (as main effects) were significant. The regression coefficient for Simulated Eye Height was 0.11 m (95% CI = [0.07 m; 0.15 m]), that is, across all postures, the lower eye height was related to 0.11 m lower PSEs than the medium eye height, and the higher eye height was related to 0.11 m higher PSEs in comparison to the medium eye height. Higher PSEs meant that the square was set larger, which in turn means that it was perceived as smaller.

For the interaction between Simulated Eye height and Posture, we found that the Sitting Posture increased the PSE gain induced by Simulated Eye height by 0.06 m (95% CI = [0.03 m; 0.09 m]) in comparison to Standing, by 0.04 m (95% CI = [0.02 m; 0.07 m]) in comparison to Elevated, and by 0.07 m (95% CI = [0.04 m; 0.9 m]) in comparison to Lying Supine.

As an incidental result, we also found that further distances were related to higher PSEs, by 0.02 m (95% CI = [0.01 m; 0.03 m]) for each simulated meter, that is, for a target distance of 12 m, the PSEs were 0.12 m higher than for a distance of 6 m, and for a distance of 18 m the PSEs were 0.24 m higher than for a distance of 6 m.

**Figure 7:**
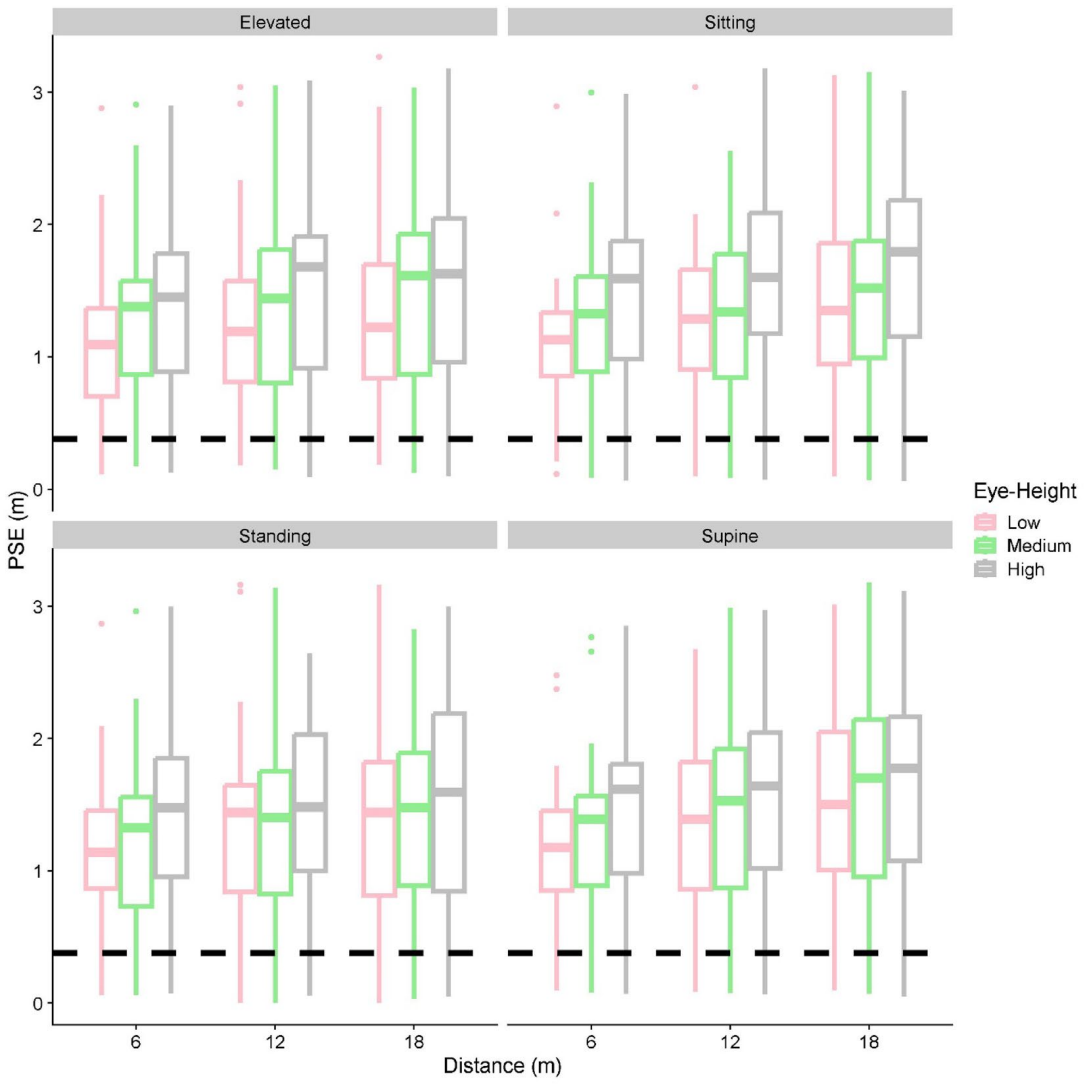
Boxplots of the PSEs, separately for each simulated distance (x axis), eye-height, color-coded; “Low” (pink), “Medium” (green) and “High” (grey) from left to right within each distance, and posture (different panels). The dashed line indicates the length of the reference stick.

**Figure 8:**
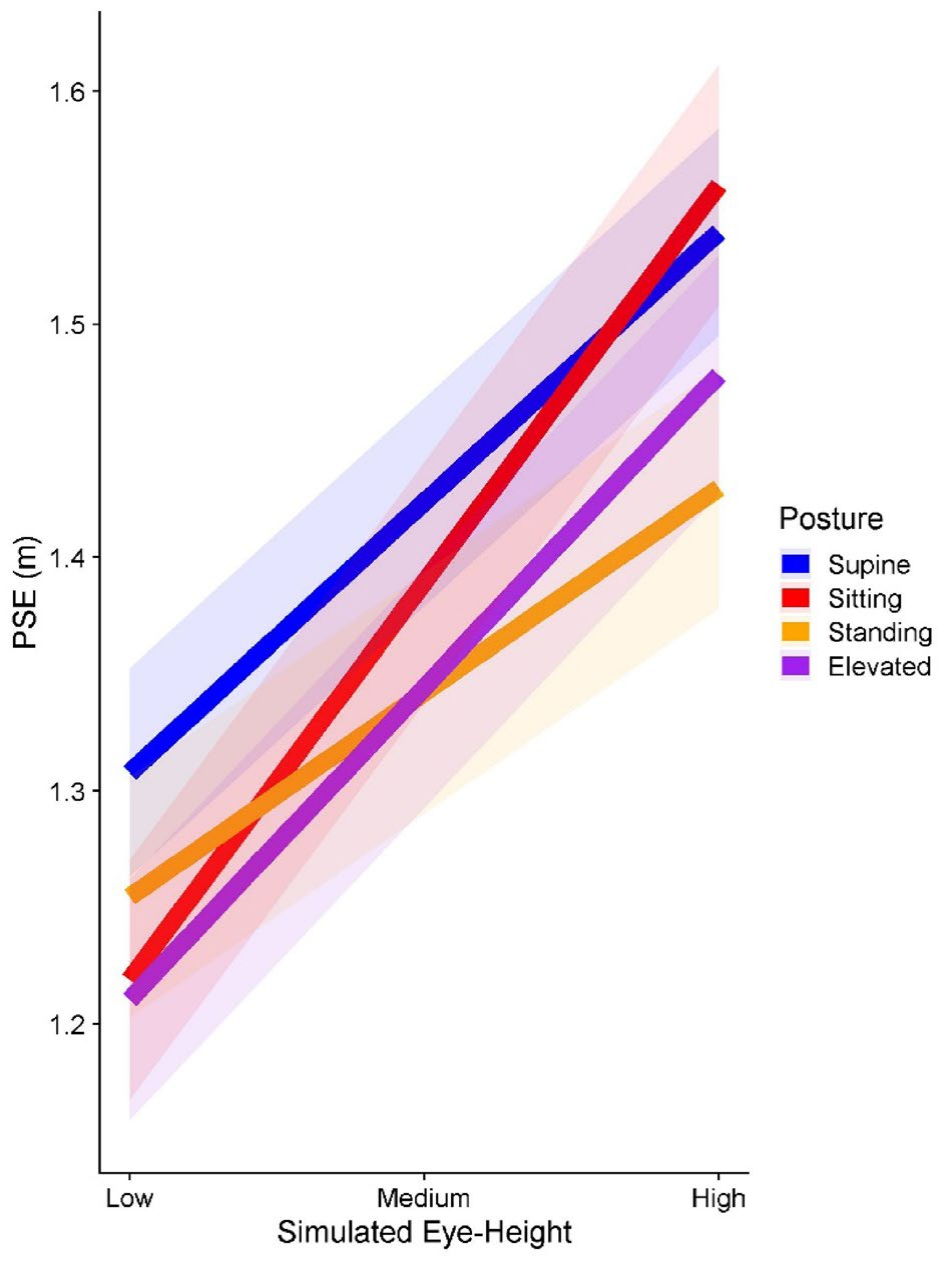
Fitted linear regression lines (using the geom_smooth() function with the method = “lm” argument from the R package ggplot^25^) with the corresponding confidence bands (± 1 standard error) for the PSEs across the three simulated eye heights, separately for each posture (color-coded). The steepness of the slopes indicates to what extent participants are affected by variation in eye height, with more shallow slopes indicating more similar performance across the three simulated eye heights, and steeper slopes indicating that performance changed more drastically from one simulated eye height to the next.

We presented these data in two ways: Fig. 7 aims to give an overview over the distributions of PSEs we obtained, while Fig. 8 illustrates the interaction between simulated eye height and posture, with a steeper slope for Sitting than for the other postures.

## Discussion

We set out to study how size perception in VR is affected by simulated eye height, by real-world postures, and the interaction between these two factors. Confirming previous results^10,12,13,15,17,21,26,27^, we found that higher simulated eye heights led participants to judge the virtual object as smaller (and thus set them larger to compensate). Further, and somewhat surprisingly given previous research^28,29^, we did not find evidence that performance differed between any of the real-world postures we tested (lying supine, sitting, standing, standing on a table). Most importantly, we found that variation in simulated eye height affected size judgements more when they were sitting than when they were lying supine, standing, or standing on a table.

### No main effect of posture

We found no main effect of posture, i.e., overall performance did not differ significantly between lying supine, sitting upright, standing, and standing on a table. A recent study^30^ investigated the effect of real-world body orientation (standing and lying supine) on perceived egocentric distance in VR. The authors used an innovative design in which they had participants compare the egocentric distance of the observer to a target in front of them to the distance between this target and secondary targets that were simulated laterally to the egocentric target, i.e., the observer, the egocentric target and the secondary formed an L shape and the participants had to compare egocentric distance to the lateral distance between the egocentric target and the secondary targets. While this study did find differences between some of the tested postures, no significant difference was found between the standing upright condition and the lying supine condition—in line with our findings.

However, our results do conflict with at least two previous studies^28,29^, which found differences between upright postures (sitting or standing) and lying supine. Harris and Mander had their participants compare the length of a stick in their hands to the length of a line projected on a wall in front of them when they were lying supine and when they were sitting upright and found that the length of the line was underestimated when lying supine relative to sitting upright. Notably, the line was projected at eye height in this experiment, that is, the geometry to infer perceived size was less complicated (see Eq. 1) than in our experiment.

The paradigm used in Kim, McManus, and Harris was even more similar to ours: participants were immersed in a virtual hallway environment and judged the height of a virtual box in this environment against the length of a reference stick held in their hands while they were standing or lying supine. The results here were comparable to the ones found earlier by Harris and Mander: participants judged the box to be less tall when lying than when standing upright. The eye height in this study was fixed at 1.65 m, which is comparable to the average medium eye height in our study. There were several smaller differences between the studies: a stick was used as a VR target rather than a square, the physical reference stick was held horizontally, shorter simulated distances were used, simulated eye heights weren’t adjusted to the participants’ individual eye heights, and the environment appeared to have been less ambiguous than ours, as evidenced by the lower variability in their data and the fact that participants judged the size of the VR target more accurately. Particularly regarding the latter, it seems likely that our experiment introduced more uncertainty and/or ambiguity, which in turn led to much higher variability and lower statistical power than Kim, McManus and Harris—a likely reason for the discrepancy in the results.

In a forthcoming study (Jörges et al., forthcoming), the same paradigm was completed by two cohorts of participants (one cohort of astronauts and one cohort of laypeople) at a fixed eye height of 1.65 m when lying supine and when sitting upright. Neither of these cohorts displayed a significant difference between the postures, and numerically the effect for the laypeople even went in the opposite direction of what Harris and Mander and Kim, McManus and Harris found. Our experiment provides a potentially unifying explanation: eye height here was simulated at 1.65 m, that is, above the real-world eye height in the sitting posture. If increases in simulated eye height make us underestimate the size of objects more when sitting than when lying supine (as indeed we report in this paper), then this might mask effects induced by posture alone as reported by Harris and Mander (where the stimulus was presented at eye height) and Kim, McManus and Harris (where eye height was simulated approximately at the average real-world standing eye height of their participants).

### Changes in eye height affect us differently depending on the real-world posture

The main, novel finding reported here is that variation in simulated eye height affected participants more when sitting than when lying supine, standing on the ground, or standing on a table; more specifically, higher simulated eye heights increased PSEs more when sitting than in the other postures (as evidenced by the steeper slope for sitting apparent in Fig. 8), indicating an increase in their underestimation of size as simulated eye height increased. There were no significant differences in how simulated eye height affected participants between the other postures. These results only partially confirm our hypotheses: we expected participants to be more affected by variation in simulated eye height for common postures where they had a clear representation of their eye height (sitting and standing) compared to when they were in unfamiliar postures (standing on the table) or postures where there was no clear way to estimate their real-world eye height (lying supine). That is, we expected steeper slopes, i.e., a stronger association between simulated eye-height and perceived object size in Fig. 8 for sitting and standing on the ground than for lying supine and standing on the table. Our hypothesis is therefore unlikely to be true as originally stated. One explanation might be that we are generally experiencing our standing eye height while moving through our environment. Being completely static while standing might put this posture in the unfamiliar/no clear reference category along with lying supine or standing on the table. Another possibility is that there is more variability in sitting eye heights that humans experience in their daily lives because sitting eye height also depends on the type of seating compared to standing eye height which is dictated only by their anatomy.

It is also important to note that these effects (both the influence of simulated eye height itself and the interaction between simulated eye height and real-world posture) might lessen or fully dissipate as virtual reality displays improve and simulated environments become more detailed. In our, and many other VR studies, visual scenes are fairly sparse, while the technology further lowers the reliability of some depth cues (e.g., notably by introducing a vergence-accommodation conflict^31–33^). Together, this requires participants (and regular users of VR) to make more use of other sources of information such as eye height or real-world posture. It stands to reason that, as VR devices and environments improve, these (sometimes inaccurate) cues might then be replaced or overridden by more veridical cues.

In line with our results, Leyrer et al.^12^ found that higher simulated eye heights led to shorter distance judgements (analogous to our lower size estimates) and vice-versa. They found this effect when participants were standing and sitting, and to a lesser extent when they were lying prone facing forwards. Given their results for the prone posture, where they report significant differences between the high and medium eye heights and between the high and low eye heights, but not between the low and medium eye heights, one might suspect that the difference is less pronounced in this posture than in the others. This would be in line with our results, where the effect of simulated eye height was stronger when participants were sitting than when they were lying. However, they did not test directly for differences in the strength of the eye height manipulation as a function of the real-world posture. Further, their participants were lying prone and facing forwards (with their line of sight parallel to the real-world ground plane), which would allow them to obtain a representation of their real-world eye height – unlike our participants who were lying supine and facing upwards.

Contrary to us, von Castell and their colleagues^21^ found no evidence that simulated eye height affected the perceived size of a virtual room differently according to their real-world posture (sitting and standing). A major difference was that participants in the von Castell study assessed the size of their environment (a virtual room they were immersed in), while our participants were judging the size of a much smaller object within the environment that we presented. Given that in real life we interact differently with the margins of an environment in which we are immersed (e.g., avoiding, surveying space) than with objects in such environment (e.g., seeking out, manipulating), it is not unreasonable to believe that different mechanisms might be at play.

### Spatial compression in VR

There are two incidental findings in our dataset that are worth exploring and which, in our opinion, can be largely traced back to the same root cause: first, we find a large underestimation of the height of the virtual object (i.e., extremely high PSEs) compared to the physical reference stick (by a factor of more than 3 on average, see Fig. 7). Second, we found that PSEs increased significantly with distance of presentation, i.e., participants underestimated the height of the virtual object more the further away it was presented. Both of these observations could be explained by a compression of perceived space, which is a well-documented finding in virtual reality^34–36^. If participants underestimated the distance to the object, then the same retinal size would correspond to a smaller physical size, which in turn would result in higher PSEs. Similarly, if this compression were to be proportional to the distance of presentation, then the effect would be numerically stronger the further away a target was presented, which is what we found in our data set. Compression of perceptual space in VR is likely in our experiment because our environment was fairly sparse without additional objects to help provide scale and/or depth information.

### High variability in responses

Another observation worth mentioning is the high between-participant variability in PSEs. It seems unlikely that participants’ perception of the size of the object would diverge this strongly in a real-world setting and speaks to our stimulus being somewhat ambiguous and/or our task being fairly hard. There are a few factors that might play a role here: first, our participants were undergraduate students who often don’t have any experience with VR simulations and are generally not trained psychophysical observers. Second, they had to translate between a physical object that they were perceiving principally in a tactile manner and a visual object in virtual reality. Further, visual depth cues were relatively sparse (see also above), which may have led some participants to interpret the visual object much closer than others. The virtual target was also presented fairly far away from the visual horizon, which makes it harder to use cues such as the horizon ratio^17^. Finally, and perhaps most importantly, we did not provide our participants with a formalized period of familiarization with the environment. While they were able to look around the VR corridor in the beginning of each block while we set them up, they were confined to one specific, fixed viewpoint and we also did not instruct them to explore the environment with their gaze in a way that would allow them to calibrate their sensory-motor system to the display. An important question in this context is whether these discrepancies between our experiment and real-life situations make it (exceedingly) hard to generalize from experiment to reality. We would argue that these sources of variability largely increase measurement error rather than providing qualitative differences to real life. They would thus make it harder to statistically detect true effects by increasing noise rather than introducing biases. However, further research – either with more veridical displays or in real life – is required before drawing firm conclusions in this regard.

### An ecological perspective

It is important to acknowledge and make explicit that this study was conceptualized from a computationalist-representationalist point of view, with its assumptions and commitments. How might we situate our results in an ecological framework, leading to a perhaps more parsimonious explanation? The (replicated) finding that perceived size generally depends on eye height can be easily explained by referring to the “horizon ratio”^17,19^, i.e., the fact that, for any but the shortest distances, the ratio between the size of an object resting on the ground and the observer’s eye height is the same as the ratio between the angle subtended by the object and the angle from the horizon to the bottom of the object. The notion that participants use their actual eye height (of which they maintain a representation) in combination with their eye height in a virtual environment in order to interpret its size, and with that the size of objects embedded in it, is therefore not necessary to explain this relationship between simulated eye height and perceive size.

The main, novel finding of this study is that humans are more influenced by variation in simulated eye height when they are sitting down than in the other postures tested in this study. One ecological, embodied explanation might be that we tend to execute different tasks when sitting than when, for example, standing or lying down. Assuming that the perceptual system is indeed geared towards the execution of specific tasks in a situated manner rather than towards maintaining representations of relevant pieces of information about the environment, this is therefore a plausible finding. However, it is not immediately clear why we would find this particular pattern: tasks executed while sitting are often more limited to the immediate peri-personal space and could more easily be accomplished using other modalities (specifically touch) than tasks executed while standing or walking, which often require planning locomotion to reach a certain target further away from the observer. Arguably, under this assumption one might then expect eye height information to be more, not less, important while standing upright. However, one could also turn this argument on its head: when sitting, eye-height information is generally less important and, therefore, the sensory-motor system would allocate fewer resources to correcting biases introduced by abnormal eye heights, therefore leading to increased biases. But, taking a step back, the absence of a difference in reliance on eye height between lying down and standing upright, which are certainly more different even than sitting and standing, shows that the difference in task context alone is inadequate to explain this finding.

From an ecological perspective, it is further important to note two characteristics of our experiment: First, our task was fairly cognitive and removed from real-life tasks and situations. It is highly likely that performance would be more precise and more accurate overall if it was more action-related, e.g., if we used grasp width as the dependent variable or even if the task required participants to judge whether our virtual object fit through (for example) a virtual door. While we are inclined to believe that the effects reported in this manuscript would hold under more naturalistic circumstances, it is certainly an empirical question to be addressed. A second relevant characteristic is VR presentation. VR presentation might obstruct or alter processes that play a role under naturalistic circumstances such as perception–action coupling or multisensory aspects of size perception. This may be a fruitful direction for future research, not only in terms of size perception but in terms of perception in VR in general.

## Conclusions

In this study, we set out to investigate whether visually simulated eye height in VR, which is known to affect the perceived size and distance of objects, exerts its effect differentially depending on the posture of the participant. We found that, indeed, higher simulated eye heights led participants to underestimate the size of a virtual box more when they were sitting upright than when they were lying supine, standing on the floor, or standing elevated on a table. Our original hypothesis, namely that a lack of a clear reference for the real-world eye height (lying supine) as well as unfamiliar real-world eye heights (standing on a table) would lead participants to be less influenced by variation in simulated eye height than in familiar postures (sitting and standing on the ground), was not fully supported because we found no difference between the standing posture and the lying supine and standing on a table postures. Further research into what makes us take into account visually simulated eye height more when sitting than in other postures is needed.

Potential applications for this line of research are principally in the development of VR technology and programming: if differences in real-world postures can lead participants to interpret simulated eye heights differently and thus be biased in their size and/or distance perception, this should be taken into account when designing VR applications and instructing participants in how to use such applications. While this is mostly nice-to-have for gaming and other entertainment applications, these observations may be critical in serious gaming using VR for training firefighters and other professions.

## Data Availability

All data and code used in this paper can be found in this OSF repository: https://osf.io/r9czu/.
